# Characterizing White Matter Changes along Fibers in Treatment-Naive Pediatric Posttraumatic Stress Disorder

**DOI:** 10.1155/2023/9020854

**Published:** 2023-05-09

**Authors:** Xueling Suo, Du Lei, Huaiqiang Sun, Wenbin Li, Kun Qin, Lingjiang Li, Graham J. Kemp, Song Wang, Qiyong Gong

**Affiliations:** ^1^Department of Radiology and Huaxi MR Research Center (HMRRC), Functional and Molecular Imaging Key Laboratory of Sichuan Province, West China Hospital, Sichuan University, Chengdu 610041, China; ^2^Research Unit of Psychoradiology, Chinese Academy of Medical Sciences, Chengdu 610041, China; ^3^Department of Psychiatry and Behavioral Neuroscience, University of Cincinnati, Cincinnati, OH 45227, USA; ^4^Department of Magnetic Resonance Imaging, The First Affiliated Hospital of Zhengzhou University, Zhengzhou 450052, China; ^5^Mental Health Institute, The Second Xiangya Hospital of Central South University, Changsha 410008, China; ^6^Liverpool Magnetic Resonance Imaging Centre (LiMRIC) and Institute of Life Course and Medical Sciences, University of Liverpool, Liverpool L69 3GE, UK; ^7^Department of Radiology, West China Xiamen Hospital of Sichuan University, Xiamen 361022, China

## Abstract

Children and adolescents are more susceptible than adults to developing posttraumatic stress disorder (PTSD). Pediatric PTSD is characterized by functional alterations in brain fear circuitry, but little is known about the underlying microstructural changes; previous work has mainly focused on the corpus callosum. This study is aimed at investigating brain-wide microstructural abnormalities in pediatric PTSD, their relationship to age and sex, and their potential diagnostic value. The microstructure of major white matter tracts was assessed from diffusion tensor images acquired from 24 treatment-naive non-comorbid PTSD patients <18 years and 24 trauma-exposed non-PTSD controls (TENP) matched for age, sex, and years of education. Statistical analyses included pointwise comparisons, correlations with symptom severity, and diagnosis-by-age/sex interactions; support vector machine analyses were conducted to determine whether microstructure distinguishes PTSD from TENP. Compared with TENP, pediatric PTSD patients showed higher fractional anisotropy and lower radial diffusivity in right superior longitudinal fasciculus and lower axial diffusivity in right uncinate fasciculus. These white matter microstructural abnormalities were highly correlated with PTSD symptom severity. No significant diagnosis by age or sex interaction was observed. The pointwise axial diffusivity measurements presented the best PTSD vs. TENP classification performance. In summary, pediatric PTSD patients showed clinically relevant microstructural abnormalities in uncinate and superior longitudinal fasciculus, which extend understanding of pediatric PTSD neurobiology beyond the corpus callosum and have diagnostic potential in distinguishing stressed individuals with and without PTSD.

## 1. Introduction

Pediatric posttraumatic stress disorder (PTSD) is a debilitating condition affecting 5% of under-18s [1], leading to functional impairment [2], sleep disturbances [3], and mood and anxiety disorders [4]. From the perspective of the emerging field of psychoradiology [5–7], better knowledge of the underlying neural substrate allows development of objective biomarkers that could assist in early identification of the condition or in forecasting its onset or course.

Most neuroimaging studies of PTSD have been of adults [8], and extrapolation is not straightforward to the distinctive pediatric variation [9], in this population undergoing extraordinary neurodevelopment, particularly in white matter [10]. Most studies of white matter in pediatric PTSD have focused on the largest fiber bundle, the corpus callosum, reporting some microstructural abnormalities [11–13]. However, few studies have examined the broader system of white matter tracts. Moreover, conventional analyses of whole tract mean diffusion measures may miss important information, as tissue properties can vary along a tract [14]. A recent study of pediatric PTSD found that the uncinate fasciculus, inferior longitudinal fasciculus, and cingulum bundle showed remarkable age- and sex-linked microstructural changes [15]; however, the comparison to nontraumatized healthy controls obscured whether these were related to PTSD *per se* or a general consequence of traumatic stress exposure [15], and there were potential confounding effects of psychiatric comorbidity and medication.

To overcome these limitations, we aimed to evaluate white matter organization in pediatric patients with PTSD and trauma-exposed non-PTSD controls (TENP) using a fiber-tract segmentation approach, which investigates microstructure across 20 major white matter tracts [16]. No participants had received psychiatric medication, and none had psychiatric comorbidity. Given the previous evidence of microstructural disruption [11–13], we hypothesized (i) that PTSD would show disrupted microstructural organization in comparison to TENP and (ii) that these would be related to symptom severity. Furthermore, given possible differential effects of age and sex on white matter microstructure [15], we (iii) searched for sex/age-by-PTSD diagnosis interactions. Finally, as white matter microstructure has been shown to have diagnostic value in brain disorders [17, 18], we hypothesized (iv) that microstructural measures could serve as markers to differentiate individuals with PTSD from those with TENP.

## 2. Materials and Methods

### 2.1. Participants

A severe earthquake occurred in Sichuan Province of China in 2008, and survivors were recruited between January 2009 and August 2009 and selected through a large-scale survey of 4200 individuals using the PTSD checklist (PCL), a self-report measure with 17 items [19]. At follow-up visits 8-15 months after the earthquake, the Clinician-Administered PTSD Scale (CAPS) [20] and the Structured Clinical Interview for DSM-IV Diagnosis (SCID) [21] were used to confirm the presence/absence of a diagnosis of PTSD. Detailed inclusion and exclusion criteria are in the Supplementary Materials (available here). In brief, all participants were <18 years; the PTSD group were individuals with PCL score ≥ 35 and CAPS score ≥ 50 when the diagnosis of PTSD was confirmed by SCID interview; TENP controls were those individuals who scored <35 on PCL but did not have a diagnosis of PTSD following SCID. The final study groups were 24 treatment-naive pediatric PTSD patients without psychiatric comorbidities and 24 demographically matched stressed controls who did not develop PTSD. In this way, survivors with PTSD and those without PTSD had similar demographic characteristics and earthquake experiences.

This study was approved by the Research Ethics of West China Hospital of Sichuan University. Written informed consent was obtained from each child's guardian after the study procedures were fully explained.

### 2.2. Data Acquisition

The MRI data were acquired using a 3T MRI system with an eight-channel phase array head coil (EXCITE; General Electric) and included diffusion tensor image (DTI) and high-resolution T1 scans. MRI scanning of survivors took place 10-15 months after the earthquake, on the same day as clinical assessment. Foam padding and earplugs were used to minimize head motion and scanner noise. The diffusion sensitizing gradients were applied along 15 noncollinear directions (*b* value = 1000 s/mm^2^) together with an acquisition without diffusion weighting (*b* = 0). Imaging parameters were repetition time (TR)/echo time (TE) 12000/71.6 ms, matrix 128 × 128, field of view (FOV) 24 × 24 cm^2^, 50 slices, slice thickness 3 mm no gap, and number of excitations 2. A high-resolution T1-weighted 3D spoiled gradient recall sequence was acquired with these scanning parameters: TR/TE 8.5/3.4 ms, inversion time 400 ms, flip angle 12°, matrix size 256 × 256, FOV 24 × 24 cm^2^, 156 axial slices, and slice thickness 1 mm with no gap. The acquired MRI images were evaluated by an experienced neuroradiologist to verify image quality.

For each raw DTI dataset, after excluding those with conspicuous head motion and signal dropout, head motion was calculated using online code (https://thewinnower.com/papers/3525-a-guide-to-quantifying-head-motion-in-dti-tudies) to exclude participants with head movements of >2° rotation around the *x*, *y*, *z* directions or >2 mm displacement/translation in the *x*, *y*, *z* directions. All participants' head motion was within these criteria, and there were no significant between-group differences in head motion (details in Table S1).

### 2.3. Imaging Processing and Automatic Tracts Identification

We used FSL (FMRIB Software Library, http://www.fmrib.ox.ac.uk/fsl) for routine DTI processing, including head motion and eddy current correction, brain extraction, and tensor model fitting. Then, we used automated fiber quantification (AFQ) software [14] (https://github.com/jyeatman/AFQ) to identify 20 white matter tracts in each participant in the following steps [22]: whole-brain deterministic fiber tractography, waypoint region of interest-based tract segmentation, and probability map-based fiber refinement using the Johns Hopkins University white matter template (http://neuro.debian.net/pkgs/fsl-jhu-dti-whitematter-atlas.html). Tracts included the left and right anterior thalamic radiation, corticospinal tract, cingulum cingulate, cingulum hippocampus, inferior frontooccipital fasciculus, superior and inferior longitudinal fasciculus, uncinate, arcuate fasciculus, the forceps minor of the genu, and the forceps major of the splenium of the corpus callosum. After tract identification, the diffusion measures including fractional anisotropy (FA), mean diffusivity (MD), axial diffusivity (AD), and radial diffusivity (RD) along the tract core, defined as the tract profile, were extracted from each fiber tract. Tracts were smoothed using a 10-point moving average filter to reduce local variation caused by imaging noise.

### 2.4. Statistical Analysis

Tract profiles were compared between PTSD and TENP in a pointwise manner. Each participant's tract profile was arranged into a single matrix, which was then fed into FSL Randomize program for permutation-based statistical analysis with 10000 permutations. The statistical results were subject to familywise error correction for multiple comparisons following threshold-free cluster enhancement and thresholded at *P* < 0.05. A flowchart of pointwise comparison can be found in our earlier report [23].

Two-way analysis of variance (ANOVA) was used to analyze PTSD diagnosis-by-sex and diagnosis-by-age (dividing the distribution into younger and older groups, 10–12 and 13–16 years) interaction; *post hoc* contrasts evaluated the simple main effects if there were statistically significant interactions; the main effect of sex was tested in the whole sample and in the PTSD and TENP groups separately. We examined the relationship between CAPS scores and the mean tract profiles derived from the portion of the tracts with significant between-group differences by partial correlations, controlling for age and sex to eliminate their potential confounding effect.

To examine whether tract profiles could differentiate between PTSD patients and TENP controls, exploratory support vector machine (SVM) analyses were conducted, details of which can be found in the Supplementary Materials.

## 3. Results

### 3.1. Group Comparison of Demographics and Clinical Symptoms

Table 1 summarizes the demographic and clinical characteristics of the 24 treatment-naive PTSD patients (14 females and 10 males) and 24 TENP controls (14 females and 10 males). No significant differences in sex, age, years of education, or time since trauma were observed between the two groups (*P* > 0.05).

### 3.2. Pointwise Comparison of Tract Profile Alterations in PTSD and TENP

**Figure 1:**
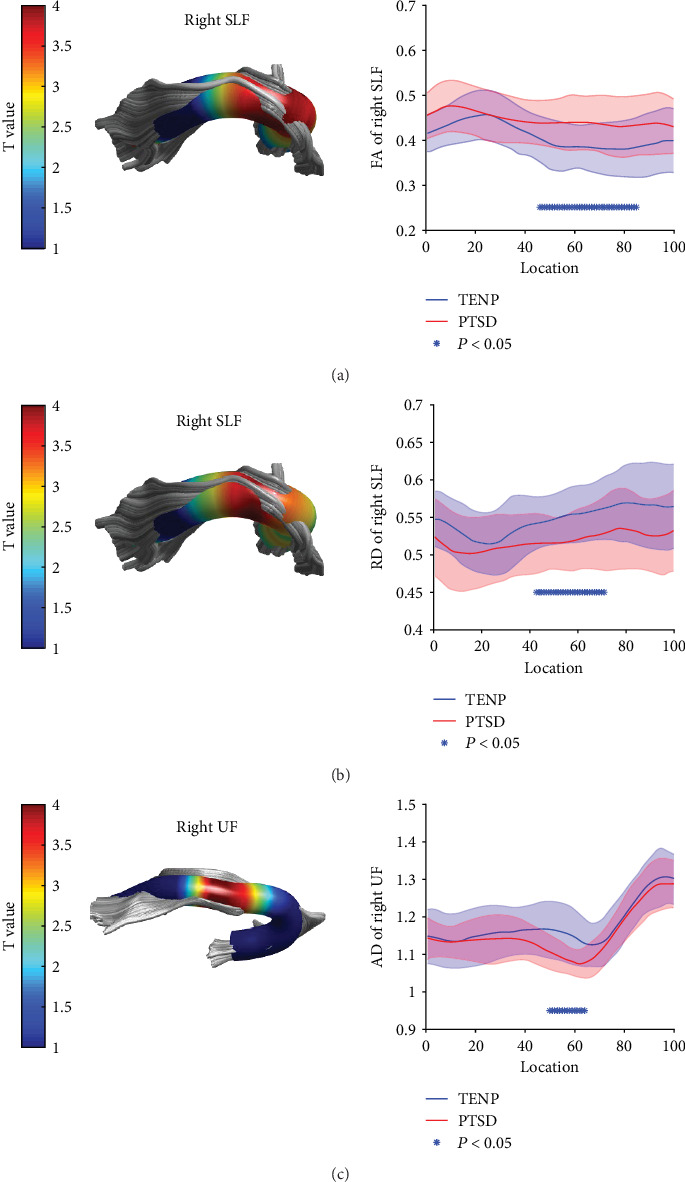
Pointwise comparison of white matter tract profiles between PTSD patients and TENP controls. For each of three tracts as labelled (namely, (a) and (b) right SLF and (c) right UF), the left-hand panel shows a 3D rendering derived from automated fiber tract quantification software for a single representative participant (color-coded for *t* value as in the key); the right-hand panel shows the corresponding group tract profiles (blue for TENP, red for PTSD; solid lines show means, shaded areas represent 1 SD). In the right-hand panels, each tract was divided into 100 equal segments (*x*-axis) and tract profiles (*y*-axis) and scaled in the same way across tracts; the asterisk bars indicate tract regions with pairwise white matter profiles significantly different between TENP controls and PTSD patients; the *y*-axis label identifies the parameter; the *x*-axis represents the location between the beginning and termination waypoint regions of interest, following the Johns Hopkins University white matter template convention. PTSD: posttraumatic stress disorder; TENP: trauma-exposed non-PTSD control; FA: fractional anisotropy; RD: radial diffusivity; AD: axial diffusivity; SLF: superior longitudinal fasciculus; UF: uncinate fasciculus.

**Figure 2:**
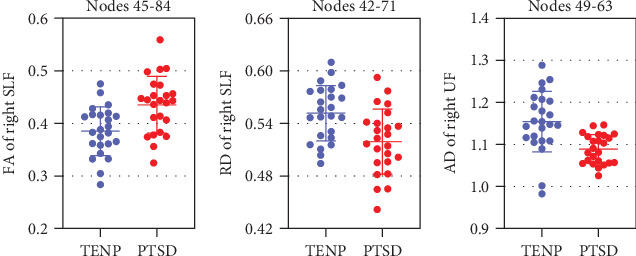
Tract segment profiles differing between PTSD patients and TENP controls. Data are shown as individual data points and mean values plus 1-SD error bars for the three tract segments (see labels on each graph) showing significant group differences between PTSD patients and TENP controls. PTSD: posttraumatic stress disorder; TENP: trauma-exposed non-PTSD control; FA: fractional anisotropy; RD: radial diffusivity; AD: axial diffusivity; SLF: superior longitudinal fasciculus; UF: uncinate fasciculus.

Figures S1–S4 show the tract profiles for each group.  Figure 1 and Table 2 show the tract segments that showed significant between-group differences in tract profiles (FWE correction, *P* < 0.05). For the right superior longitudinal fasciculus, PTSD patients relative to TENP controls showed significantly higher FA in points 45-84 (Figure 1(a)) and lower RD in points 42-71 (Figure 1(b)); for the right uncinate fasciculus, PTSD patients relative to TENP showed lower AD in points 49-63 (Figure 1(c)). No significant between-group differences were observed in MD.  Figure 2 shows mean values of individual data points for the tract segments that showed significant group differences.

### 3.3. Interaction between Groups and Age/Sex in terms of Tract Profiles

Two-way ANOVA revealed no significant diagnosis-by-age or diagnosis-by-sex interaction in FA (*P* = 0.07 and 0.07) or RD (*P* = 0.99 and 0.65) in the right superior longitudinal fasciculus or AD (*P* = 0.61 and 0.61) in the right uncinate fasciculus. Tables S2–S5 show the main effect of sex on brain white matter in the whole sample and within each group. Briefly, in the combined group only, male relative to female participants showed significantly higher MD in right inferior longitudinal fasciculus, with no significant between-group sex differences in other fiber tracts or within either group.

### 3.4. Correlations between Alterations in White Matter Measures and Clinical Symptoms

**Figure 3:**
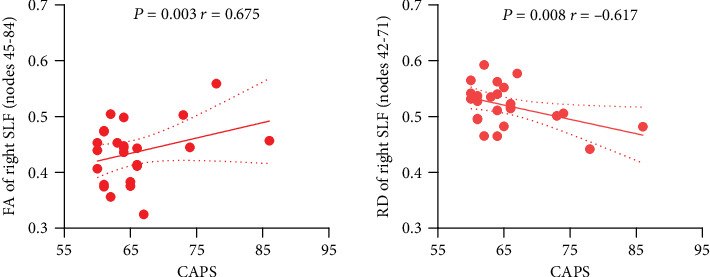
Relationships between white matter tract profile alterations and CAPS scores in PTSD. The graphs show the relationship between the white matter tract profile alterations (i.e., mean values from the nodes significantly different between groups: parameter and location given in *y*-axis label) and CAPS scores. The graphs show lines of regression (solid) and 95% error lines (dotted). PTSD: posttraumatic stress disorder; CAPS: Clinician-Administered PTSD Scale; FA: fractional anisotropy; RD: radial diffusivity; SLF: superior longitudinal fasciculus.

Figure 3 shows the negative correlation between CAPS score and mean RD over the nodes 42-71 of right superior longitudinal fasciculus (*P* = 0.008, *r* = −0.617) and the positive correlation between CAPS scores and mean FA over nodes 45-84 of right superior longitudinal fasciculus (*P* = 0.003, *r* = 0.675). There was no significant correlation between CAPS score and mean AD over 49-63 nodes of right uncinate fasciculus.

### 3.5. Classification Performance

Table S6 shows the results of SVM for diagnosis (PTSD vs. TENP) based on tract profiles. The mean balanced accuracies of classifications were all above chance, the best performer being the pointwise AD profile (accuracy 70.8%; sensitivity 62.7%; specificity 79.0%).

## 4. Discussion

We applied fiber-tract segmentation to identify localized white matter tract microstructural abnormalities in treatment-naive pediatric PTSD patients compared to TENP controls and to investigate their potential utility in diagnosis. We found significantly higher FA and lower RD in right superior longitudinal fasciculus and lower AD in right uncinate fasciculus; altered white matter profiles in those tracts were correlated with clinical symptom severity, but showed no significant group-age or group-sex interactions, and the pointwise AD profile performed best in classifying TENP controls and PTSD patients. This study adds to our understanding of the white matter microstructure in pediatric PTSD and might help in developing imaging-based diagnostic tools.

Consistent with previous studies [24, 25], we found lower AD in the right uncinate fasciculus in PTSD patients. As current neurobiological models of PTSD emphasize frontotemporal connectivity, it is not surprising that effects on microstructural organization include the uncinate fasciculus, which connects the orbitofrontal and the anterior temporal cortices related to memory formation and emotion regulation [26]. A recent case study used deep brain stimulation in the uncinate fasciculus to successfully treat a PTSD patient, supporting a causal role and suggesting a novel targeting strategy [27]. We also found higher FA and lower RD in the right superior longitudinal fasciculus. This large tract connects the prefrontal cortex with the parietal, occipital, and temporal lobes and is associated with a range of functions including perception of visual and auditory space and aspects of motor behavior [28]. The simple frontotemporal model of PTSD would not predict effects on the superior longitudinal fasciculus, but it has emerged among areas affected in meta-analysis [11, 13] and may be predictive of PTSD treatment efficacy in victims of interpersonal violence [29]. In PTSD, it perhaps functions in broader aspects of visual-spatial attention in information processing and autobiographical memory.

That uncinate fasciculus and superior longitudinal fasciculus findings were predominantly lateralized to the right hemisphere is consistent with reported abnormalities of right-sided cerebral perfusion, electroencephalographic, magnetoencephalography synchrony, and cortical excitability in PTSD associated with intrusive emotional autobiographical memory, arousal, avoidance, and reexperience [30–33]. However, reports of lateralized hemispheric deficits in PTSD conflict [34, 35] and the relation may be more indirect [36]. More research is needed on this. No significant group-age or group-sex interactions in terms of tract profiles were observed in the current study. However, diagnosis-by-sex/age interaction has been recently found in the white matter study of pediatric PTSD [15]. This discrepancy may be explained by differences in study design, such as the type of control group (TENP vs. nontraumatized healthy controls), type and duration of trauma, medication status, and comorbidities [12]. Technical factors of DTI acquisition and analysis may also contribute.

In our study, the main contribution to higher FA was lower RD. RD has been proposed as an *in vivo* marker of myelin, lower RD being associated with increased myelination [37]. There is neuroimaging evidence of increased myelination in adult PTSD [38, 39], but this has not been previously shown in pediatric PTSD. Rat models of stress show increased oligodendrogenesis, a precursor to myelin synthesis [40]. It may be that the lower RD we observed reflects adaptive oligodendrogenesis and myelination, and that this provides a structural basis for observations of altered neural dynamics in functional MRI studies in PTSD [41, 42]. However, these RD changes should be interpreted with caution.

Although machine learning is not yet routinely available, given the urgent need for early-stage disease biomarkers, the evidence reported here is promising for the development of diagnostic markers based on imaging. Our multivariate classification and cross-validation suggests that white matter profiles may be useful for individual diagnosis of PTSD. We believe this is the first use of machine learning to examine this potential of white matter tract profiles: our exploratory SVM analyses identified the AD profiles as discriminating PTSD from non-PTSD with 70% accuracy and 79% specificity, giving mean balanced accuracy convincingly above chance. Several other PTSD studies have applied machine learning to other kinds of neuroimaging data: pretreatment resting-state functional connectivity achieved 76% accuracy predicting trauma-focused psychotherapy response for individual young PTSD patients [43]; hippocampal subfield volumes with random forest classifiers achieved 69% accuracy in distinguishing traumatized children with and without PTSD [44]; a deep learning model based on graph-theoretic features achieved 89% accuracy discriminating adult PTSD patients from TENP controls [45]. Despite this potential, in none of these examples is accuracy particularly high. Better classification performance may be achieved using other advanced models [46], although computation involving large numbers of parameters will require a larger sample size. Whether our findings are specific to PTSD, rather than transdiagnostic characteristics of many psychiatric disorders, needs to be explored in future studies.

Our study has limitations. First, the cross-sectional design limits inferences about individual microstructural changes over time and prediction of PTSD conversion after major life stress and precluded exploration of the relationship between PTSD and neurodevelopment. Longitudinal follow-up design in future studies would help clarify these issues. Second, our focus was on white matter microstructures that differentiate between similarly stressed individuals with and without PTSD. Without a comparison group of nontraumatized healthy controls, we cannot examine the effects of general stress [47, 48]. This limits the potential for clinical application, and further comprehensive studies in clinical settings are needed. Third, all participants in our study were exposed to a single type of trauma; one should be cautious about generalizing to PTSD due to other types of trauma. Fourth, the performance of the SVM classification model was not very high; other advanced machine learning techniques such as deep learning may improve classification performance, although these usually involve higher levels of complexity and computation of more parameters, requiring a much larger sample size. Fifth, while it provides information about white matter microstructure, the detection of group differences may have been limited by the relatively small sample size resulting from our strict recruitment criteria. Developing a comprehensive model of white matter microstructure will require larger PTSD pediatric samples, but adhering to stringent criteria to maintain a sufficiently homogenous subject group.

Notwithstanding these limitations, this work makes a novel contribution to knowledge of pediatric PTSD's neural basis. We have confirmed white matter microstructure abnormalities, widespread beyond the corpus callosum [15]. By comparing survivors with and without PTSD, we have identified these as related to PTSD specifically, rather than simply to traumatic stress. Finally, by controlling for confounding variables such as psychiatric medication and comorbidity, we have identified changes related, as far as possible, to the underlying disease process. Previous studies have found that PTSD after different kinds of trauma may have different cerebral deficits [49, 50]. The present study may therefore be particularly useful in improving the reliability and specificity of biomarkers to identify the single-stressor PTSD caused by natural disasters.

## 5. Conclusion

Combining a tractography approach with SVM analyses, our study found abnormal diffusion measures of fiber tracts in pediatric PTSD patients with potential diagnostic utility. We found abnormal microstructural organization in right uncinate fasciculus and right superior longitudinal fasciculus tracts, which were related to symptom severity. Our findings support white matter abnormalities as a promising diagnostic marker in pediatric PTSD and throw light on white matter changes beyond the corpus callosum.

## Figures and Tables

**Table 1:** Demographics and clinical characteristics of the participants^a^.

Variables	TENP (*n* = 24)	PTSD (*n* = 24)	*P* value
Age (years)^b^	12.9 ± 1.2	13.2 ± 1.8	0.57^c^
Sex (male/female)	10/14	10/14	1.00^d^
Years of education^b^	7.8 ± 2.0	7.8 ± 1.7	0.95^c^
Time since trauma (months)^b^	12.3 ± 1.8	11.4 ± 1.3	0.07^c^
PTSD checklist	24.1 ± 2.8	55.0 ± 5.3	<0.001^c^
CAPS		65.5 ± 6.3	

^a^Data are presented as the mean ± SD (minimum-maximum) unless noted. ^b^Age, years of education, and time since trauma were defined relative to the time of MRI scanning. ^c^*P* value by two-sample two-tailed *t*-test. ^d^*P* value by two-tailed chi-squared test. PTSD: posttraumatic stress disorder; TENP: trauma-exposed non-PTSD; CAPS: Clinician-Administered PTSD Scale.

**Table 2:** Regions across the 20 fiber tracts where white matter measures differ significantly between PTSD patients and TENP controls.

Diffusion measures/fiber tracts	Location	TENP	PTSD
Fractional anisotropy (FA)			
Right superior longitudinal fasciculus	Nodes 45-84	0.385 ± 0.046	0.435 ± 0.054
Mean diffusivity (MD)	No regions of difference		
Radial diffusivity (RD)			
Right superior longitudinal fasciculus	Nodes 42-71	0.552 ± 0.031	0.519 ± 0.037
Axial diffusivity (AD)			
Right uncinate fasciculus	Nodes 49-63	1.154 ± 0.072	1.089 ± 0.035

Measurements are presented as the mean ± SD. Regions were considered abnormal if they exhibited significant between-group differences (*P* < 0.05) using permutation-based analysis (10000 permutations) in FSL Randomize. Nodes are defined by dividing each tract into 100 equal segments between the beginning and termination waypoint regions of interest along the given tract. PTSD: posttraumatic stress disorder; TENP: trauma-exposed non-PTSD controls.

## Data Availability

The data that support the findings of this study are available from the corresponding authors upon reasonable request.

## Supplementary Materials

Supplementary Materials Supplementary Methods and Materials. Figure S1: pointwise comparison of fractional anisotropy (FA) between TENP and PTSD. Figure S2: pointwise comparison of axial diffusivity (AD) between TENP and PTSD. Figure S3: pointwise comparison of mean diffusivity (MD) between TENP and PTSD. Figure S4: pointwise comparison of radial diffusivity (RD) between TENP and PTSD. Table S1: comparison of head movements between PTSD and TENP. Table S2: sex differences of fractional anisotropy in all subjects and each group. Table S3: sex differences of axial diffusivity in all subjects and each group. Table S4: sex differences of mean diffusivity in all subjects and each group. Table S5: sex differences of radial diffusivity in all subjects and each group. Table S6: single-subject classification of PTSD patients and TENP controls. References: the supplementary materials has been provided by the authors to give readers additional information about their work.
