# Supplementary material for: Characterizing White Matter Changes along Fibers in Treatment-Naive Pediatric Posttraumatic Stress Disorder
Source: Depress Anxiety. 2023 May 9;2023:9020854. doi: 10.1155/2023/9020854 (PMC11921854; doi:10.1155/2023/9020854)
Supplement: Supplementary Materials — Supplementary Methods and Materials. Figure S1: pointwise comparison of fractional anisotropy (FA) between TENP and PTSD. Figure S2: pointwise comparison of axial diffusivity (AD) between TENP and PTSD. Figure S3: pointwise comparison of mean diffusivity (MD) between TENP and PTSD. Figure S4: pointwise comparison of radial diffusivity (RD) between TENP and PTSD. Table S1: comparison of head movements between PTSD and TENP. Table S2: sex differences of fractional anisotropy in all subjects and each group. Table S3: sex differences of axial diffusivity in all subjects and each group. Table S4: sex differences of mean diffusivity in all subjects and each group. Table S5: sex differences of radial diffusivity in all subjects and each group. Table S6: single-subject classification of PTSD patients and TENP controls. References: the supplementary materials has been provided by the authors to give readers additional information about their work. [file 9020854.f1.docx]

# **Supplementary Materials**

# **Methods and Materials**

## **Participants**

The following inclusion criteria applied for all participants: (i) age < 18 years; (ii) right-handed; (iii) physical experience of the earthquake; (iv) personal witness of building collapse, death or serious injury; (v) no known PTSD prior to the earthquake; and (vi) no psychologic interventions or psychopharmacologic treatment before MRI. Exclusion criteria were: age ≥ 18 years; left-handed; reported serious traumatic events before or after the earthquake, current and lifetime psychiatric comorbidities such as depression and anxiety disorders, or alcohol/drug/other substance abuse/dependence; traumatic brain injury; neurological or cardiovascular conditions; any contraindication to MRI; brain lesions identified at MRI examination.

## **Support vector machine (SVM) analyses**

Exploratory SVM analyses were applied to the tract profiles of FA, MD, AD and RD to determine whether tract profile can distinguish PTSD patients and TENP controls at the individual level. The SVM model maps the input vectors to a feature space using a set of mathematical functions known as kernels ([Cortes and Vapnik, 1995](#_ENREF_1)). In this feature space, the model finds the optimum separation surface that maximizes the margin of separation between different classes within a training dataset. Once the separation surface is determined, it can be used to predict the class of new observations using an independent testing dataset. Here a linear kernel was preferred to a nonlinear one to minimize the risk of overfitting. The model was based on LIBSVM and implemented using the Scikit-Learn library ([Pedregosa et al., 2011](#_ENREF_4)). A 5-fold stratified cross-validation was used, dividing the original sample into 5 nonoverlapping folds that preserved the relative proportion of the two classes. In each iteration four folds were defined as the training set and the remaining fold as the test set. The linear SVM has only one parameter (soft margin parameter C), which controls the trade-off between reducing training errors and increasing the separation margin. An internal cross-validation was performed to select the optimal parameter, by performing a grid search (C = 10^-3^, 10^-2^, 10^-1^, 10^0^, 10^1^, 10^2^, 10^3^, 10^4^) to estimate the best value. An SVM model with the optimal parameter was trained on the training set, and its performance was assessed on the test set in terms of balanced accuracy, specificity, and sensitivity. The reported values are means calculated on each partition of the cross-validation scheme. To estimate the significance for each SVM, a nonparametric permutation test were performed to calculate a P value for balanced accuracy ([Golland and Fischl, 2003](#_ENREF_2)). This involved repeating the classification procedure 1000 times with different random permutations of the group labels. A P value was then calculated by dividing the number of times that the balanced accuracy was higher for the permuted labels than the real labels by 1000. For more detail, please refer to our recent report ([Lei et al., 2020](#_ENREF_3)).

**
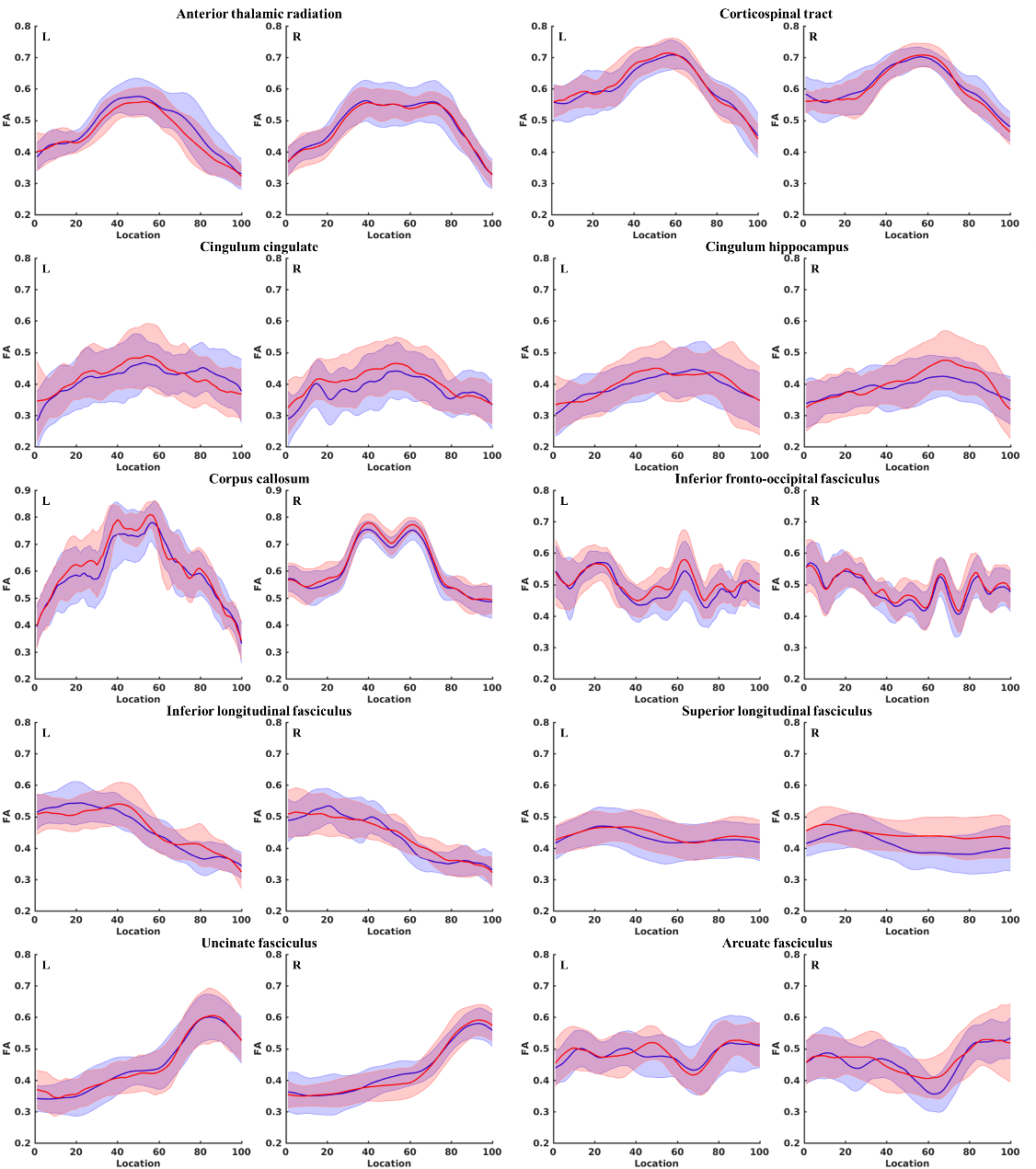
**

**Figure S1. Point-wise comparison of fractional anisotropy (FA) between TENP and PTSD**

The FA profiles are color-coded (blue for TENP, red for PTSD; solid lines show means, shaded areas represent SDs). Pairs of panels show results from a named tract, left and right. Each tract was divided into 100 equal segments (X axis) and FA profiles (Y axis) and scaled in the same way across tracts. The x-axis represents the location between the beginning and termination waypoint regions of interest along the given tract. Abbreviations: L, left; R, right; PTSD, posttraumatic stress disorder; TENP, trauma-exposed non-PTSD.


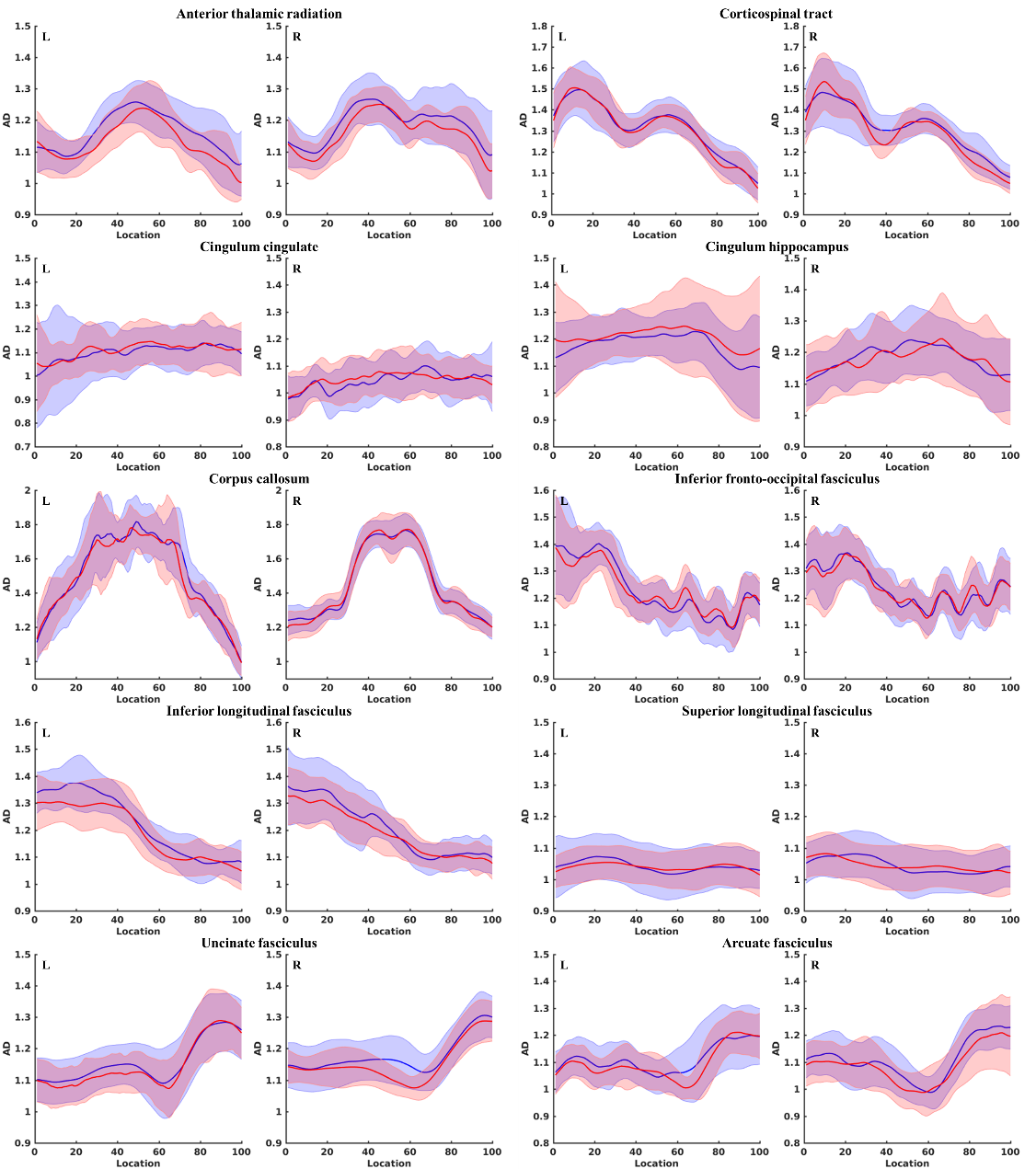


**Figure S2. Point-wise comparison of axial diﬀusivity (AD) between TENP and PTSD**

The AD profiles are color-coded (blue for TENP, red for PTSD; solid lines show means, shaded areas represent SDs). Pairs of panels show results from a named tract, left and right. Each tract was divided into 100 equal segments (X axis) and AD profiles (Y axis) and scaled in the same way across tracts. The x-axis represents the location between the beginning and termination waypoint regions of interest along the given tract. Abbreviations: L, left; R, right; PTSD, posttraumatic stress disorder; TENP, trauma-exposed non-PTSD.

**
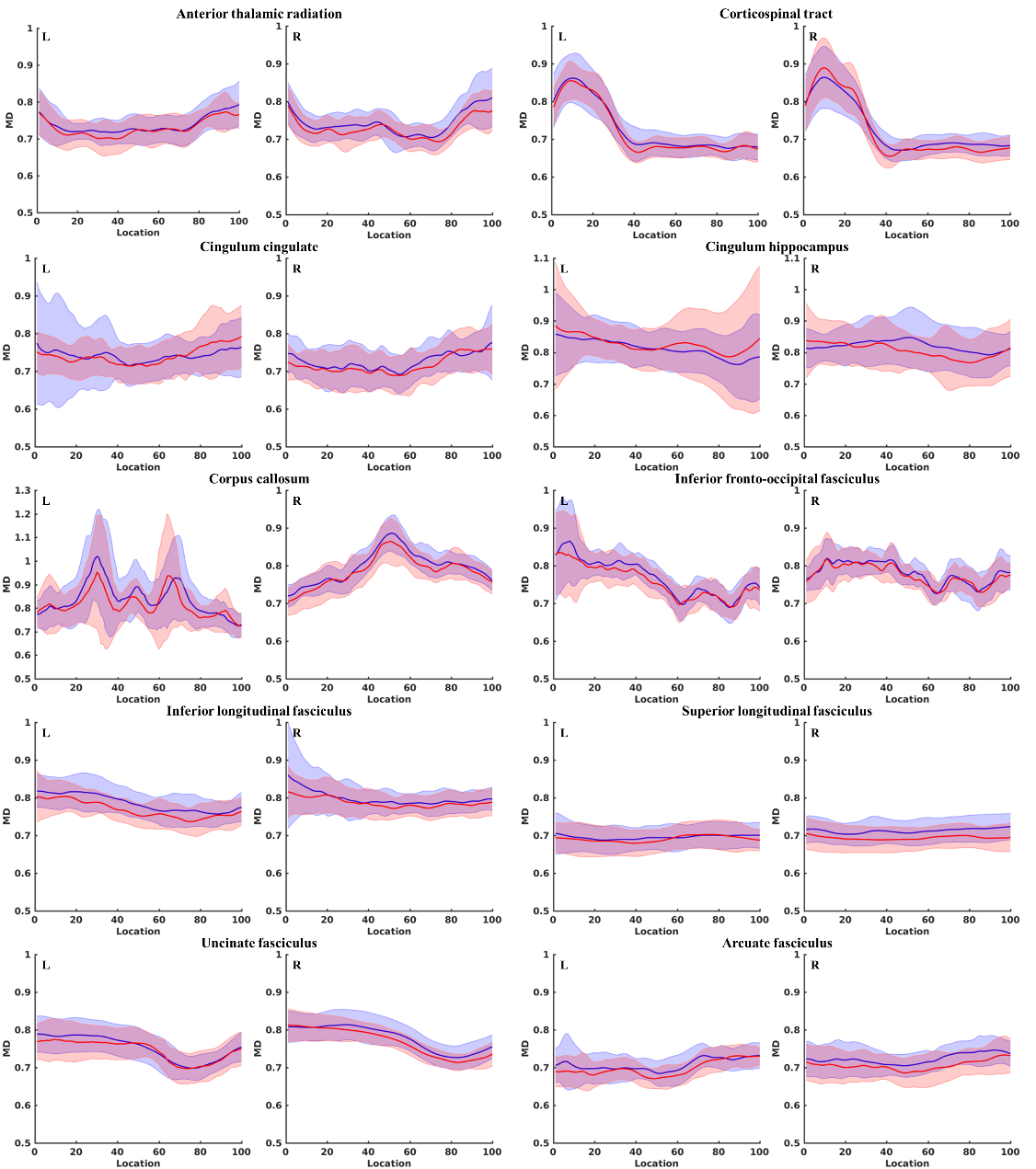
**

**Figure S3. Point-wise comparison of mean diﬀusivity (MD) between TENP and PTSD**

The MD profiles are color-coded (blue for TENP, red for PTSD; solid lines show means, shaded areas represent SDs). Pairs of panels show results from a named tract, left and right. Each tract was divided into 100 equal segments (X axis) and MD profiles (Y axis) and scaled in the same way across tracts. The x-axis represents the location between the beginning and termination waypoint regions of interest along the given tract. Abbreviations: L, left; R, right; PTSD, posttraumatic stress disorder; TENP, trauma-exposed non-PTSD.


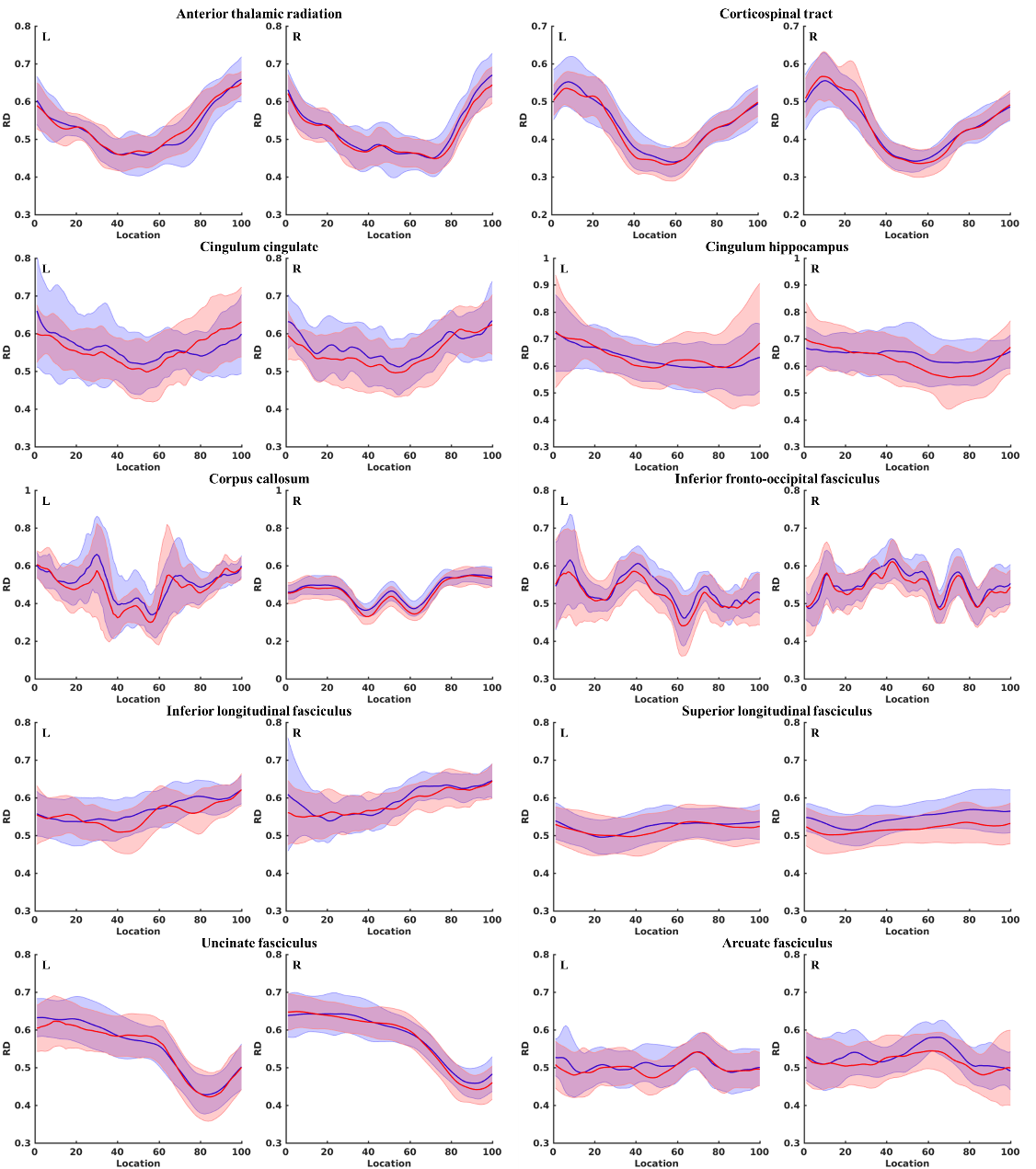


**Figure S4. Point-wise comparison of radial diﬀusivity (RD) between TENP and PTSD**

The RD profiles are color-coded (blue for TENP, red for PTSD; solid lines show means, shaded areas represent SDs). Pairs of panels show results from a named tract, left and right. Each tract was divided into 100 equal segments (X axis) and RD profiles (Y axis) and scaled in the same way across tracts. The x-axis represents the location between the beginning and termination waypoint regions of interest along the given tract. Abbreviations: L, left; R, right; PTSD, posttraumatic stress disorder; TENP, trauma-exposed non-PTSD.

**Table S1. Comparison of head movements between PTSD and TENP**

| Measurements | TENP ^a^ | PTSD ^a^ | *P* ^b^ |
| --- | --- | --- | --- |
| Relative displacement | 0.546 ± 0.118 | 0.554 ± 0.077 | 0.766 |
| Rotation around x | 0.002 ± 0.004 | 0.0004 ± 0.005 | 0.209 |
| Rotation around y | -0.001 ± 0.002 | -0.001 ± 0.003 | 0.527 |
| Rotation around z | 0.0002 ± 0.001 | -0.0005 ± 0.002 | 0.126 |
| Translation along x | -0.030 ± 0.050 | -0.020 ± 0.081 | 0.602 |
| Translation along y | 0.219 ± 0.149 | 0.259 ± 0.157 | 0.363 |
| Translation along z | 0.285 ± 0.464 | 0.404 ± 0.285 | 0.276 |

^a^ Data are mean ± standard deviation.

^b^ *P* value by two-sample two-tailed t test.

Abbreviations: PTSD, posttraumatic stress disorder; TENP, trauma-exposed non-PTSD.

**Table S2. Sex differences of fractional anisotropy in all subjects and each group**

|  | All subjects | |  | TENP | |  | PTSD | |
| --- | --- | --- | --- | --- | --- | --- | --- | --- |
|  | *t* | *P* |  | *t* | *P* |  | *t* | *P* |
| Left thalamic radiation | 1.426 | 0.161 |  | 0.861 | 0.399 |  | 1.214 | 0.238 |
| Right thalamic radiation | 0.962 | 0.341 |  | 1.306 | 0.205 |  | -0.173 | 0.864 |
| Left corticospinal tact | 1.314 | 0.195 |  | -0.437 | 0.666 |  | 2.544 | 0.018 |
| Right corticospinal tact | 1.224 | 0.227 |  | 0.047 | 0.963 |  | 1.830 | 0.081 |
| Left cingulum cingulate | -2.12 | 0.039 |  | -1.359 | 0.188 |  | -1.573 | 0.130 |
| Right cingulum cingulate | -0.121 | 0.904 |  | 0.697 | 0.493 |  | -0.765 | 0.452 |
| Left cingulum hippocampus | -0.412 | 0.682 |  | -1.419 | 0.170 |  | 0.525 | 0.605 |
| Right cingulum hippocampus | -0.782 | 0.438 |  | -1.939 | 0.065 |  | 0.532 | 0.600 |
| Left corpus callosum | -0.871 | 0.388 |  | 0.507 | 0.617 |  | -1.705 | 0.102 |
| Right corpus callosum | 0.22 | 0.827 |  | 0.606 | 0.550 |  | -0.308 | 0.761 |
| Left inferior fronto-occipital fasciculus | -0.879 | 0.384 |  | -0.702 | 0.490 |  | -0.570 | 0.575 |
| Right inferior fronto-occipital fasciculus | -1.776 | 0.082 |  | -1.062 | 0.300 |  | -1.406 | 0.174 |
| Left inferior longitudinal fasciculus | -1.032 | 0.308 |  | -1.599 | 0.124 |  | -0.111 | 0.913 |
| Right inferior longitudinal fasciculus | -0.077 | 0.939 |  | 0.174 | 0.863 |  | -0.253 | 0.803 |
| Left superior longitudinal fasciculus | -0.321 | 0.750 |  | -0.432 | 0.670 |  | 0.067 | 0.948 |
| Right superior longitudinal fasciculus | 0.782 | 0.438 |  | 1.915 | 0.069 |  | -0.408 | 0.687 |
| Left uncinate fasciculus | 1.318 | 0.194 |  | 0.370 | 0.715 |  | 1.360 | 0.188 |
| Right uncinate fasciculus | 1.612 | 0.114 |  | 0.943 | 0.356 |  | 1.274 | 0.216 |
| Left arcuate fasciculus | 0.263 | 0.793 |  | 1.405 | 0.174 |  | -0.927 | 0.364 |
| Right arcuate fasciculus | -0.378 | 0.707 |  | 0.213 | 0.833 |  | -0.615 | 0.545 |

Tracts were considered abnormal if they showed significant male-female differences (*P* < 0.05/20, familywise error corrected).

Abbreviations: PTSD, posttraumatic stress disorder; TENP, trauma-exposed non-PTSD.

**Table S3. Sex differences of axial diﬀusivity in all subjects and each group**

|  | All subjects | |  | TENP | |  | PTSD | |
| --- | --- | --- | --- | --- | --- | --- | --- | --- |
|  | *t* | *P* |  | *t* | *P* |  | *t* | *P* |
| Left thalamic radiation | 1.743 | 0.088 |  | 1.992 | 0.059 |  | 0.687 | 0.499 |
| Right thalamic radiation | 1.975 | 0.054 |  | 2.260 | 0.034 |  | 0.426 | 0.674 |
| Left corticospinal tact | 2.322 | 0.025 |  | 1.574 | 0.130 |  | 1.703 | 0.103 |
| Right corticospinal tact | 2.832 | 0.007 |  | 1.815 | 0.083 |  | 2.358 | 0.028 |
| Left cingulum cingulate | 0.128 | 0.899 |  | 0.050 | 0.961 |  | 0.151 | 0.881 |
| Right cingulum cingulate | 1.291 | 0.203 |  | 1.588 | 0.127 |  | 0.057 | 0.955 |
| Left cingulum hippocampus | 0.363 | 0.718 |  | 0.930 | 0.362 |  | -0.208 | 0.837 |
| Right cingulum hippocampus | 1.699 | 0.096 |  | 0.430 | 0.671 |  | 2.071 | 0.050 |
| Left corpus callosum | 2.947 | 0.005 |  | 1.877 | 0.074 |  | 2.221 | 0.037 |
| Right corpus callosum | 1.41 | 0.165 |  | 1.536 | 0.139 |  | 0.433 | 0.669 |
| Left inferior fronto-occipital fasciculus | 1.42 | 0.162 |  | 1.022 | 0.318 |  | 0.946 | 0.355 |
| Right inferior fronto-occipital fasciculus | 1.197 | 0.237 |  | 0.519 | 0.609 |  | 1.198 | 0.244 |
| Left inferior longitudinal fasciculus | 1.344 | 0.186 |  | 1.393 | 0.178 |  | 0.572 | 0.573 |
| Right inferior longitudinal fasciculus | 2.98 | 0.005 |  | 2.072 | 0.050 |  | 2.212 | 0.038 |
| Left superior longitudinal fasciculus | 2.351 | 0.023 |  | 0.892 | 0.382 |  | 2.942 | 0.008 |
| Right superior longitudinal fasciculus | 1.679 | 0.100 |  | 1.658 | 0.111 |  | 0.655 | 0.519 |
| Left uncinate fasciculus | 0.267 | 0.79 |  | -0.746 | 0.464 |  | 1.442 | 0.163 |
| Right uncinate fasciculus | 0.551 | 0.584 |  | 0.396 | 0.696 |  | 0.443 | 0.662 |
| Left arcuate fasciculus | 2.141 | 0.038 |  | 2.355 | 0.028 |  | 0.502 | 0.620 |
| Right arcuate fasciculus | 0.766 | 0.448 |  | 1.259 | 0.221 |  | 0.005 | 0.996 |

Tracts were considered abnormal if they showed significant male-female differences (*P* < 0.05/20, familywise error corrected).

Abbreviations: PTSD, posttraumatic stress disorder; TENP, trauma-exposed non-PTSD.

**Table S4. Sex differences of mean diﬀusivity in all subjects and each group**

|  | All subjects | |  | TENP | |  | PTSD | |
| --- | --- | --- | --- | --- | --- | --- | --- | --- |
|  | *t* | *P* |  | *t* | *P* |  | *t* | *P* |
| Left thalamic radiation | 0.822 | 0.415 |  | 1.268 | 0.218 |  | -0.124 | 0.903 |
| Right thalamic radiation | 1.163 | 0.251 |  | 1.148 | 0.263 |  | 0.532 | 0.600 |
| Left corticospinal tact | 1.452 | 0.153 |  | 2.159 | 0.042 |  | -0.394 | 0.697 |
| Right corticospinal tact | 2.293 | 0.026 |  | 2.387 | 0.026 |  | 0.841 | 0.409 |
| Left cingulum cingulate | 1.477 | 0.146 |  | 0.529 | 0.602 |  | 1.810 | 0.084 |
| Right cingulum cingulate | 1.153 | 0.255 |  | 0.833 | 0.414 |  | 0.779 | 0.444 |
| Left cingulum hippocampus | 0.707 | 0.483 |  | 1.861 | 0.076 |  | -0.525 | 0.605 |
| Right cingulum hippocampus | 1.802 | 0.078 |  | 1.359 | 0.188 |  | 1.171 | 0.254 |
| Left corpus callosum | 2.589 | 0.013 |  | 1.199 | 0.243 |  | 2.366 | 0.027 |
| Right corpus callosum | 1.16 | 0.252 |  | 1.234 | 0.230 |  | 0.531 | 0.601 |
| Left inferior fronto-occipital fasciculus | 2.835 | 0.007 |  | 2.000 | 0.058 |  | 2.053 | 0.052 |
| Right inferior fronto-occipital fasciculus | 3.177 | 0.003 |  | 1.626 | 0.118 |  | 2.955 | 0.007 |
| Left inferior longitudinal fasciculus | 2.402 | 0.020 |  | 2.877 | 0.009 |  | 0.823 | 0.419 |
| Right inferior longitudinal fasciculus | 3.326 | 0.002* |  | 2.406 | 0.025 |  | 2.309 | 0.031 |
| Left superior longitudinal fasciculus | 2.593 | 0.013 |  | 1.165 | 0.256 |  | 2.549 | 0.018 |
| Right superior longitudinal fasciculus | 1.349 | 0.184 |  | 0.494 | 0.626 |  | 1.523 | 0.142 |
| Left uncinate fasciculus | -1.054 | 0.297 |  | -1.206 | 0.241 |  | -0.328 | 0.746 |
| Right uncinate fasciculus | -0.897 | 0.374 |  | -0.328 | 0.746 |  | -1.123 | 0.273 |
| Left arcuate fasciculus | 2.407 | 0.020 |  | 1.498 | 0.148 |  | 2.009 | 0.057 |
| Right arcuate fasciculus | 0.911 | 0.367 |  | 0.810 | 0.427 |  | 0.536 | 0.597 |

*Tracts were considered abnormal if they showed significant male-female differences (*P* < 0.05/20, familywise error corrected).

Abbreviations: PTSD, posttraumatic stress disorder; TENP, trauma-exposed non-PTSD.

**Table S5. Sex differences of radial diﬀusivity in all subjects and each group**

|  | All subjects | |  | TENP | |  | PTSD | |
| --- | --- | --- | --- | --- | --- | --- | --- | --- |
|  | *t* | *P* |  | *t* | *P* |  | *t* | *P* |
| Left thalamic radiation | -0.172 | 0.864 |  | 0.352 | 0.728 |  | -0.642 | 0.527 |
| Right thalamic radiation | 0.024 | 0.981 |  | -0.358 | 0.724 |  | 0.447 | 0.660 |
| Left corticospinal tact | -0.119 | 0.906 |  | 1.583 | 0.128 |  | -2.012 | 0.057 |
| Right corticospinal tact | 0.603 | 0.550 |  | 1.615 | 0.121 |  | -0.540 | 0.595 |
| Left cingulum cingulate | 2.042 | 0.047 |  | 0.803 | 0.431 |  | 2.147 | 0.043 |
| Right cingulum cingulate | 0.671 | 0.506 |  | 0.122 | 0.904 |  | 0.817 | 0.423 |
| Left cingulum hippocampus | 0.815 | 0.419 |  | 2.194 | 0.039 |  | -0.648 | 0.524 |
| Right cingulum hippocampus | 1.447 | 0.155 |  | 1.653 | 0.112 |  | 0.484 | 0.633 |
| Left corpus callosum | 1.81 | 0.077 |  | 0.263 | 0.795 |  | 2.086 | 0.049 |
| Right corpus callosum | 0.419 | 0.677 |  | 0.150 | 0.882 |  | 0.434 | 0.668 |
| Left inferior fronto-occipital fasciculus | 2.753 | 0.008 |  | 2.494 | 0.021 |  | 1.712 | 0.101 |
| Right inferior fronto-occipital fasciculus | 3.055 | 0.004 |  | 2.000 | 0.058 |  | 2.312 | 0.031 |
| Left inferior longitudinal fasciculus | 2.077 | 0.043 |  | 2.770 | 0.011 |  | 0.552 | 0.586 |
| Right inferior longitudinal fasciculus | 2.183 | 0.034 |  | 1.804 | 0.085 |  | 1.307 | 0.205 |
| Left superior longitudinal fasciculus | 1.689 | 0.098 |  | 0.812 | 0.426 |  | 1.557 | 0.134 |
| Right superior longitudinal fasciculus | 0.445 | 0.658 |  | -0.709 | 0.486 |  | 1.247 | 0.226 |
| Left uncinate fasciculus | -1.458 | 0.152 |  | -1.050 | 0.305 |  | -1.007 | 0.325 |
| Right uncinate fasciculus | -1.472 | 0.148 |  | -0.763 | 0.453 |  | -1.275 | 0.215 |
| Left arcuate fasciculus | 1.23 | 0.225 |  | 0.017 | 0.986 |  | 1.634 | 0.116 |
| Right arcuate fasciculus | 0.605 | 0.548 |  | 0.233 | 0.818 |  | 0.584 | 0.565 |

*Tracts were considered abnormal if they showed significant male-female differences (*P* < 0.05/20, familywise error corrected).

Abbreviations: PTSD, posttraumatic stress disorder; TENP, trauma-exposed non-PTSD.

**Table S6. Single-subject classification of PTSD patients and TENP controls**

| **Measures** | **Accuracy (%)** | **Sensitivity ^a^ (%)** | **Specificity ^a^ (%)** |
| --- | --- | --- | --- |
| AD | 70.8 ± 9.5 | 62.7 ± 17.2 | 79.0 ± 2.0 |
| FA | 68.0 ± 9.8 | 56.0 ± 29.4 | 80.0 ±17.9 |
| MD | 68.8 ± 10.4 | 66.7 ± 22.3 | 71.0 ±20.1 |
| RD | 69.7 ± 9.3 | 59.3 ± 26.3 | 80.0 ± 17.9 |

^a^ Sensitivity and specificity were computed taking PTSD as the positive class. Results are mean ± standard deviation.

Abbreviations: PTSD, posttraumatic stress disorder; TENP, trauma-exposed non-PTSD; FA, fractional anisotropy; AD, axial diffusivity; MD, mean diffusivity; RD, radial diffusivity.

# **References**

Cortes, C., Vapnik, V. (1995) Support-vector networks. Mach Learn, 20, 273-297. <http://dx.doi.org/10.1007/BF00994018>

Golland, P., Fischl, B. (2003) Permutation tests for classification: towards statistical significance in image-based studies. Inf Process Med Imaging, 18, 330-341. <http://dx.doi.org/10.1007/978-3-540-45087-0_28>

Lei, D., Pinaya, W. H. L., van Amelsvoort, T., Marcelis, M., Donohoe, G., Mothersill, D. O., Corvin, A., Gill, M., Vieira, S., Huang, X., Lui, S., Scarpazza, C., Young, J., Arango, C., Bullmore, E., Qiyong, G., McGuire, P., Mechelli, A. (2020) Detecting schizophrenia at the level of the individual: relative diagnostic value of whole-brain images, connectome-wide functional connectivity and graph-based metrics. Psychol Med, 50, 1852-1861. <http://dx.doi.org/10.1017/S0033291719001934>

Pedregosa, F., Varoquaux, G., Gramfort, A., Michel, V., Thirion, B., Grisel, O., Blondel, M., Prettenhofer, P., Weiss, R., Dubourg, V. (2011) Scikit-learn: Machine learning in Python. J Mach Learn Res, 12, 2825-2830.
